# Plant plasma membrane-bound staphylococcal-like DNases as a novel class of eukaryotic nucleases

**DOI:** 10.1186/1471-2229-12-195

**Published:** 2012-10-26

**Authors:** Krzysztof Leśniewicz, Elżbieta Poręba, Michalina Smolarkiewicz, Natalia Wolff, Sławoj Stanisławski, Przemysław Wojtaszek

**Affiliations:** 1 Department of Molecular and Cellular Biology, Institute of Molecular Biology and Biotechnology, Adam Mickiewicz University, Poznań, 89 Umultowska St, Poznan 61-614, Poland; 2 Department of Molecular Virology, Institute of Experimental Biology, Adam Mickiewicz University, Poznań, 89 Umultowska St, Poznan, 61-614, Poland

**Keywords:** Staphylococcal-like nuclease, Programmed cell death, Plasma-membrane protein, Senescence, Myristoylation/palmitylation motif, ABC transporter, tRNA synthetase

## Abstract

**Background:**

The activity of degradative nucleases responsible for genomic DNA digestion has been observed in all kingdoms of life. It is believed that the main function of DNA degradation occurring during plant programmed cell death is redistribution of nucleic acid derived products such as nitrogen, phosphorus and nucleotide bases. Plant degradative nucleases that have been studied so far belong mainly to the S1-type family and were identified in cellular compartments containing nucleic acids or in the organelles where they are stored before final application. However, the explanation of how degraded DNA components are exported from the dying cells for further reutilization remains open.

**Results:**

Bioinformatic and experimental data presented in this paper indicate that two *Arabidopsis* staphylococcal-like nucleases, named CAN1 and CAN2, are anchored to the cell membrane via N-terminal myristoylation and palmitoylation modifications. Both proteins possess a unique hybrid structure in their catalytic domain consisting of staphylococcal nuclease-like and tRNA synthetase anticodon binding-like motifs. They are neutral, Ca^2+^-dependent nucleaces showing a different specificity toward the ssDNA, dsDNA and RNA substrates. A study of microarray experiments and endogenous nuclease activity revealed that expression of CAN1 gene correlates with different forms of programmed cell death, while the CAN2 gene is constitutively expressed.

**Conclusions:**

In this paper we present evidence showing that two plant staphylococcal-like nucleases belong to a new, as yet unidentified class of eukaryotic nucleases, characterized by unique plasma membrane localization. The identification of this class of nucleases indicates that plant cells possess additional, so far uncharacterized, mechanisms responsible for DNA and RNA degradation. The potential functions of these nucleases in relation to their unique intracellular location are discussed.

## Background

Deoxyribonucleases are a large group of enzymes characterized by considerable structural and functional diversity. In eukaryotic cells they are involved in a range of cellular functions, including DNA repair, recombination and genome degradation. The degradation of nuclear DNA, a hallmark of programmed cell death (PCD), is a process that occurs both in animals and in plants. However, despite the many functional similarities between plant and animal PCD, degradation of nuclear DNA in representatives of these two kingdoms seems to serve fundamentally different purposes. DNA is an immunogenic molecule for animals and if the DNA of apoptotic cells is left undigested it can cause inflammatory responses and auto-immune diseases [1]. In plants, where these processes do not occur, degradation of DNA as well as RNA is most likely involved in nutrient remobilization, since nucleic acids are a rich source of nitrogen and phosphorus, which belong to the major limiting nutrients for plant growth [2,3].

The known degradative nucleases involved in plant PCD belong mainly to the S1 family, identified as sharing a sequence similarity with the S1 nuclease from *Aspergillus oryzae*[4]. One of the best characterized members of this family, BFN1 endonuclease, is induced during senescence and developmental PCD of *Arabidopsis*[5,6]. Direct evidence of nuclease function in PCD has been reported for another member of this family, ZEN1, which is responsible for DNA degradation during xylogenesis [7]. Moreover, the expression of other S1 type nucleases has been identified in various tissues undergoing PCD [8,9].

The expression, activity and cellular location of degradative nucleases are strictly controlled due to their potential toxicity to host cells. In animals apoptotic DNA degradation occurs in two systems. In first, DNA endonucleolytical hydrolysis is carried out by caspase-activated DNase (CAD), which in non-apoptotic cells resides in the nucleus as an inactive enzyme bound with an inhibitor protein (DFF45). The caspase-dependent apoptotic pathway releases CAD nuclease, which is further activated by specific chromosomal proteins [1]. Moreover, mitochondrial caspase-independent endonuclease G, seems to be also involved in apoptotic DNA cleavage [10]. In the next stage, the fragmented DNA is digested by lysosomal DNaseII after the dying cells are phagocytosed.

It has been proposed that in plants the degradative nucleases, as well as other hydrolytic enzymes involved in PCD, accumulate in the vacuole of maturing cells. Then, during the final stage of PCD, tonoplast rupture releases them into the cytoplasm resulting in autolysis of the cell contents [11]. Although such a mechanism has been observed during tracheary element programmed cell death [12], Farage-Barhom *et al.*[13] reported that the senescence-associated BFN1 nuclease is initially deposited in filamentous structures spread throughout the cytoplasm but in late senescent cells, it was localized in fragmented nuclei vesicles. It should be noted that, although the endonucleolytic cleavage of nuclear DNA in plant cells undergoing PCD has been well-documented, the explanation of how degraded DNA components are exported from the dying cells for further reutilization remains open.

Degradative nucleases are also used by some bacteria to utilize exogenous DNA molecules as a nutrient source [14]. Although most of these enzymes are secretory proteins, the membrane-associated nucleases that have been identified in various mycoplasma species are particularly interesting in the context of this paper. Mycoplasma is an intracellular bacterium which is devoid of enzymes involved in the biosynthesis of nucleotides. However, mycoplasmas are able to acquire nucleotides from host nucleic acids. It has been shown that some mycoplasma nucleases can be considered pathogenic determinants because of their ability to induce the apoptotic changes characterized by the internucleosomal fragmentation of host chromatin [15]. Furthermore, other observations have revealed that some mycoplasma membrane-associated nucleases are components of an ABC transport system involved in the import of nucleotides released from host DNA [16]. These mycoplasma nucleases are processed for attachment to the cell membrane through the addition of a lipid moiety. Amino acid sequence analysis revealed that known mycoplasma membrane-associated nucleases possess one of the two types of catalytic domains, i.e. DNaseI-like motif in the case of *mnuA* nuclease of *M. pulmonis*[17] or staphylococcal-like SNc domain in mph379 [16] and MG_186 [18].

The staphylococcal nuclease (SNc) domain was first described for the Nuc thermonuclease of *Staphylococcus aureus*, in which this protein is secreted to degrade exogenous nucleic acids. Characterized bacterial SNase domain containing proteins are small, heat stable, Ca^2+^-dependent nucleases, digesting single-stranded DNA and/or double stranded DNA [19]. Proteins containing staphylococcal nuclease domain(s) have also been identified in other kingdoms of life. The Tudor staphylococcal nucleases (TSNs), the best characterized multifunctional eukaryotic proteins of this class, are composed of tandem repeats of staphylococcal nuclease-like domains [20]. The next group of plant proteins showing similarity to staphylococcal nuclease is characterized by the presence of a single SNase domain. In the *Arabidopsis* genome, this small family consists of two genes, i.e. At3g56170 and At2g40410. The literature concerning this protein family is very limited. Isono *et al.*[21] and Guo *et al.*[22] reported that the bacterially expressed protein products of *Arabidopsis* staphylococcal-like genes, named the CAN proteins (Calcium dependent nuclease), reveal DNase activity. Moreover, Gu *et al.*[23] observed that the expression of the cucumber homologue of the *Arabidopsis* CAN nuclease, designated CsCaN, is ethylene inducible and is probably involved in the primordial anther-specific DNA damage of developing female cucumber flowers.

In this paper we present experimental evidence showing that two plant staphylococcal-like nucleases belong to a new, as yet unidentified class of eukaryotic nucleases, characterized by unique plasma membrane localization. Despite their sequence similarity, both of these enzymes show different catalytic properties and expression profiles, suggesting that they might exert different biological functions. Since one of them is specifically expressed in tissues undergoing various types of PCD we suspect that it is involved in plant genomic degradation. We discuss the potential functions of these nucleases in relation to their unique intracellular location.

## Results

### CAN nucleases are predicted to be N-myristoylated and palmitoylated proteins with modified SNase domains

A BLAST search of the NCBI protein database revealed that in plants two classes of proteins possess domains homologous to active sites of staphylococcal nucleases. One of them comprises two genes, AT5G61780 and AT5G07350, containing four tandem repeats of staphylococcal nuclease-like domains followed by a tudor and C-terminal SNc domain. The second class comprises two genes, At3g56170 and At2g40410, encoding proteins designated in this paper as CAN1 and CAN2, respectively. In contrast to Tudor motive containing proteins, the CAN1 and CAN2 possess single SNase domains. Detailed analyses of both CAN amino acid sequences revealed that these proteins exhibit complex primary structures consisting of various motifs conserved throughout the different classes of proteins.

**Figure 1:**
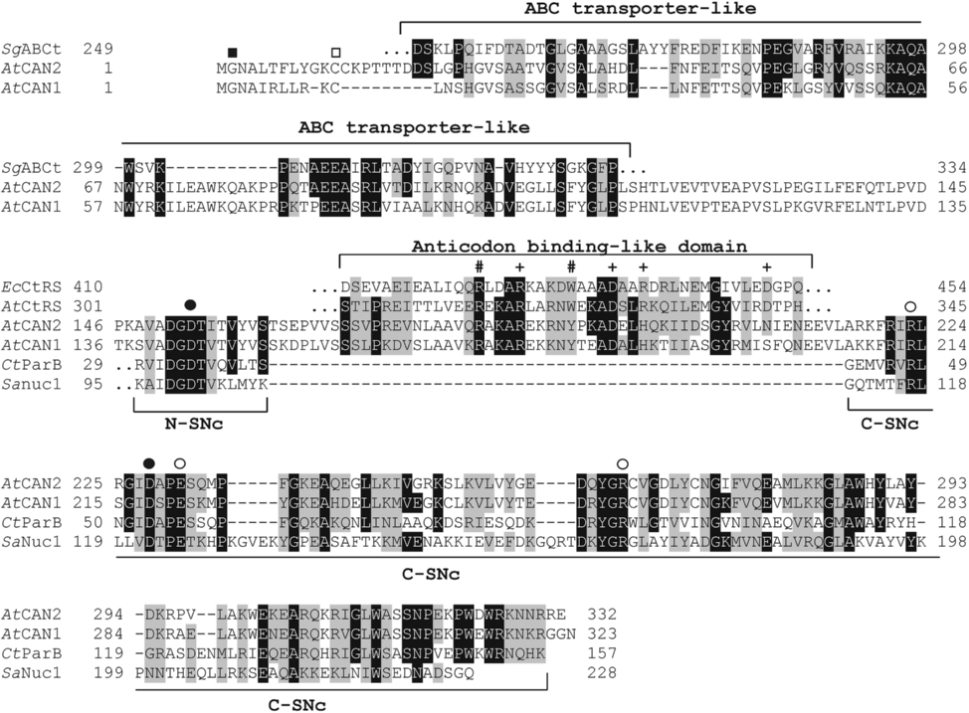
**Domain organization and sequence alignments of *Arabidopsis *CAN1 and CAN2 proteins.** The amino acid sequence alignment of two *Arabidopsis* CAN nucleases with conserved motifs of five bacterial proteins. The alignment contains the full-length sequences of CAN1 and CAN2 proteins and parts of the bacterial proteins showing the highest similarity to the corresponding regions of the CAN1 and CAN2 sequences (brackets above and below the sequences). **AtCAN1** – CAN1 nuclease of *A. thaliana* [TAIR:At3g56170]. **AtCAN2** – CAN2 nuclease of *A. thaliana* [TAIR:At2g40410]. ***Sg*ABCt** - putative periplasmic component of an ABC-type transporter of *Syntrophobotulus glycolicus* [NCBI:YP_004267097]. ***Ec*CtRS** - cysteinyl-tRNA synthetase of *Escherichia coli* [GenBank:EGW73615]. ***At*CtRS** – putative cysteinyl-tRNA synthetase of *Anaerolinea thermophila* [NCBI:YP_004174706]. ***Ct*ParB** - parB-like nuclease of *Cronobacter turicensis* [NCBI:YP_003212717]. ***Sa*Nuc1** - thermonuclease of *Staphylococcus aureus* [GenBank:EGS91983]. Identical and highly conserved amino acids are boxed in black and gray, respectively. Black square (■) - the N-terminal glycine in the putative N-myristoylation motif. Open square (□) - cysteine residues in the putative palmitoylation motif. Plus sign (+) – residues, which in cysteinyl-tRNA synthetases interact with anticodon-loop bases of bacterial tRNACys. Hash sign (#) **–** residues affecting anticodon binding stability. Black dots (**●**) - residues involved in binding of Ca^2+^ ions. Open dots (○) - residues within the active site of the SNase domain. **N-SNc** and **C-SNc** – N-terminal and C-terminal parts of a putative SNase catalytic domain, respectively.

One of the most intriguing features of both CAN proteins is the unique structure of their catalytic domains located near their carboxyl terminal ends. This domain contains all conserved amino acid residues considered to be functionally critical for the enzymatic activity of the staphylococcal nuclease (Figure 1). However, our analysis revealed that this domain is divided by a short sequence, including ca. 55 amino acids, that does not exhibit any homology to known SNase domains. This insertion separates a highly conserved N-end part of SNc domain (N-SNc), containing one of the Ca^2+^ binding aspartate residues, from the C-end part of this domain (C-SNc), containing a second Ca^2+^ binding aspartate and three amino acid residues putatively directly involved in enzyme catalysis, i.e. two arginines and one glutamate (Figure 1). Both parts of CAN1 and CAN2 SNc domains show a most significant similarity to the catalytic domain of bacterial parB nuclease; however, their similarity to well defined staphylococcal nucleases [19] is also significant. The sequence analysis of the amino acid fragment that divides two parts of SNc domain, unexpectedly revealed that this region shares homology with some bacterial cysteinyl-tRNA synthetases (Figure 1). What we find particularly interesting is that this CAN nucleases motive almost exactly corresponds to the tRNA synthetases domain responsible for the recognition of the tRNA anticodon loop [24]. Furthermore, the five out of the six residues which in tRNA synthetases either directly interact with anticodon nucleotides or are critical for the stability of the binding cavity are strictly or highly conserved in CAN proteins. It should be noted that this insertion is conserved among all plant CAN homologues including those from evolutionarily ancient plants like *Physcomitrella* and *Selaginella* (data not shown).

We also examined a potential post-translational modification of CAN nucleases. Using the Myristoylator program we identified the N-terminal myristoylation consensus motifs in both *Arabidopsis* CAN nucleases. The glycine residue at position 2, which in myristoylated proteins serves as an acceptor site for myristate [25], is also present in all other plant CAN homologues. Both CAN nucleases have been classified by Boisson *et al.*[26] to the list of 437 putatively myristoylated *Arabidopsis* proteins (N-myristoylome), determined on the basis of combined experimental and bioinformatic approaches. Myristoylation can influence the ability of a protein to interact with membrane, but the presence of myristoyl moiety is not sufficient for stable membrane attachment. It is believed that protein membrane anchorage, induced by myristoylation, is followed by palmitoylation of cysteine residue(s) [25]. Application of CSS-Palm software, designed for palmitoylation prediction [27], showed the presence of a putative palmitoylation site directly downstream of the myristoylation motive in CAN1 and CAN2 (Figure 1). Since such an arrangement of myristoylation and palmitoylation sites at the N terminus has been identified in a number of membrane proteins this suggests that this region of CAN proteins may also be involved in membrane attachment.

The CAN protein region, located between N-terminal myristoylation/palmitoylation motifs and the SNc catalytic domain does not exhibit similarities to any defined domain, neither does it reveal significant homology to any other plant or animal protein sequences. However, a BLAST search of this amino acid sequence within the protein databases showed its highest homology to some periplasmic components of bacterial ABC-type transporters [28]. Although the low sequence similarity existing between CAN nucleases and ABC transporters does not allow any definite conclusions to be drawn about their potentially analogous functions, it might indicate their putative phylogenetic relationships.

### CAN1 and CAN2 nucleases transiently expressed in protoplasts vary in their catalytic activities

The nucleotide sequences deposited in public databases have been used to design PCR primers for amplification of full length cDNAs encoding both *Arabidopsis* staphylococcal-like nucleases. The microarray data, discussed later in this article, suggest that CAN1 expression can be induced by developmental and stress cues, including age dependent senescence, and that the activity of the CAN2 gene is essentially constitutive. Consequently, we used reverse transcribed mRNA purified from early senescent leaves as a template for PCR amplification of both genes. The RT-PCR amplified products were cloned into plant expression vectors, which allowed us to obtain transient expression of recombinant native as well as C-terminal HA- and fluorescent protein-tagged fusion proteins.

The published report concerning plant staphylococcal-like nucleases demonstrated the Ca^2+^-dependent activity of bacterially expressed proteins [21,22]. However, since the activity of plant enzymes may be affected by various post-translation modifications, we decided to examine both members of this family after transient over-expression in *Arabidopsis* protoplasts. In our study we transformed the protoplasts prepared from root cell cultures and from leaf mesophile and obtained similar results.

**Figure 2:**
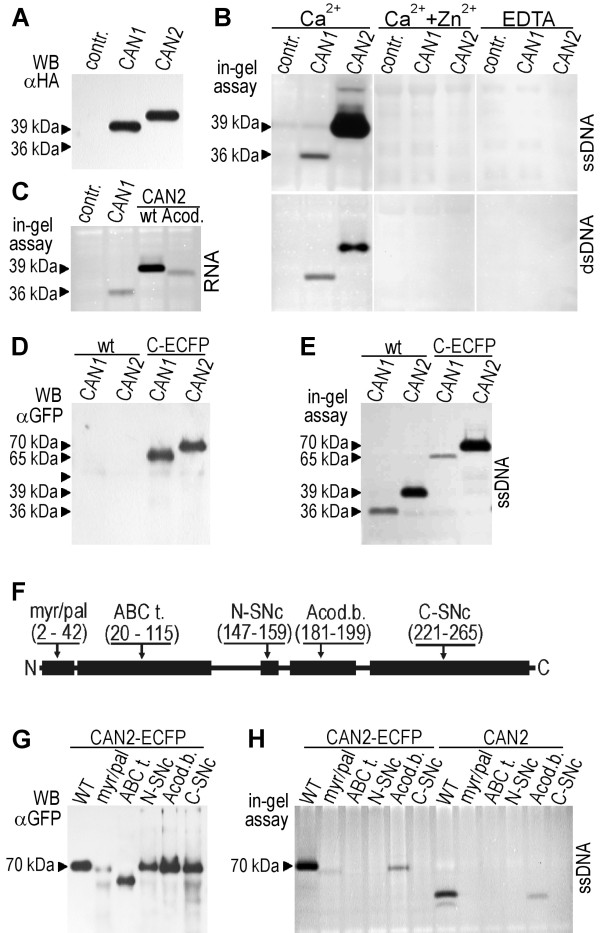
**Detection of transiently expressed CAN1 and CAN2 nuclease activity.** The protein extract from protoplasts transformed with empty vectors (pSAT6A-ECFP-N1) was used as a negative control (contr.). (**A**) Western blot analysis of HA-tagged CAN1 and CAN2 nucleases with anti-HA antibody***.*** Molecular mass deduced from gel electrophoresis as indicated by the arrowheads. (**B**) Detection of CAN1 and CAN2 DNase activities using the in-gel nuclease activity assay. Type of DNA substrate (ssDNA, dsDNA), ions and EDTA added to the reaction buffers, as indicated. (**C**) RNase activities of CAN1 wild type and CAN2 wild type (wt) and its mutant lacking anti-codon binding-like domain (Acod) are shown. **(D)** Western blot analysis of wild type (wt) and ECFP-tagged (C-ECFP) CAN1 and CAN2 proteins with anti-GFP antibody. (**E**) Detection of wild type (wt) and C-terminal ECFP-tagged (C-ECFP) CAN1 and CAN2 DNase activity using in-gel nuclease activity assay. (**F**) The deletion mutants of CAN2 lacking the conserved regions of domains described in the text. (Myr/pal.) - myristoylation and palmitoylation motif. (ABC t.) - ABC transporter-like sequence. (N-SNc) - N-terminal part of SNc domain. (C-SNc) - C-terminal part of SNc domain. (Acod.b.) - anti-codon binding-like domain. Numbers in the brackets below the domain name abbreviations indicate the ranges of amino acids removed from each mutant. (**G**) Western blot analysis of CAN2 mutants with an anti-GFP antibody. The abbreviations of the mutated domains as above (Figure 2F). (**H**) Detection of the nuclease activity of CAN2 mutants. The abbreviations of mutated domains as above (Figure 2F).

To estimate the efficiency of protein synthesis, the protein extracts from transformed protoplasts were subjected to Western blot analysis using an antibody against the HA-domain tagged at the C-terminus of the transgenes. As shown in Figure 2A, both transgenes were efficiently expressed in protoplasts prepared from *Arabidopsis* suspension cells. The molecular mass of the recombinant CAN1 and CAN2 proteins, estimated using Western blot analysis, was approximately 36 kDa and 39 kDa, roughly the same as predicted from the deduced amino acid sequences: 36.1 kDa and 37.3 kDa, respectively.

To identify the DNase activity of CAN proteins over-expressed in protoplasts, we used an in-gel nuclease activity assay. Application of this assay in combination with single stranded DNA used as a substrate and in the presence of calcium ions allowed us to detect clear bands representing nucleolytic activity at the position corresponding to the molecular mass of CAN1 and CAN2 (Figure 2B). All repeated experiments revealed that, despite the comparable efficiency of expression of both nucleases, the yield of nuclease activity was significantly higher for CAN2. Analogous experiments performed with undenatured DNA demonstrated that both nucleases also digest double stranded DNA, but in this case the activity of both nucleases is comparable. An adjustment of the reaction conditions allowed us to estimate that the maximum activity of both proteins was at pH 8.0 and a Ca^2+^ concentration of about 5 mM with a range of 1 to 10 mM (data not shown). Moreover, we found that in the presence of EDTA both nucleases did not reveal any activity. A pronounced inhibition effect was also observed with Zn^2+^. Moreover, we found that heat denaturation in the presence of 2-mercaptoethanol prior to electrophoresis does not inhibit the activities of either enzyme, suggesting a lack of the functionally important disulphide bonds in their native structure. In gel RNase assay, conducted with plant total RNA as a substrate (Figure 2C) revealed that both nucleases also have ability to degrade RNA which demonstrate that they can be defined as the sugar-nonspecific nucleases. Such a diverse substrate specificity suggests that the function of CAN nucleases is associated with degradative processes.

In the present studies we also used CAN recombinant nucleases fused with ECFP, GFP and RFP. Since the stability of recombinant proteins with a C-end or N-end attached fluorescent marker protein can be altered post-translationally by proteolytic processing we also analyzed them via western blot and in-gel nuclease activity assay. As shown in Figure 2D and E, the electrophoretic mobilities of both nucleases with C-tagged ECFP were retarded as compared with co-electrophoresed untagged proteins, corresponding to their enhanced molecular weight. Furthermore, the nuclease activity of CAN2-ECFP seems to be unaltered in comparison to untagged CAN2 and is significantly stronger than CAN1-ECFP.

Bioinformatic analysis revealed that plant CAN nucleases, in addition to SNc domains, also contain some conserved domains of unknown function. In order to understand their relevance for nucleolytic activity, the CAN2 deletion mutants were constructed to remove regions, comprising conserved residues of each domain (Figure 2F). Because we intended to use these constructs to study CAN2 nucleolytic activity as well as its cellular location, deletion mutants of untagged CAN2 and CFP-tagged CAN2 were prepared. Despite the identical condition of transformation in all repeated experiments we observed a decreased amount of CAN2 mutant with the deleted myristoylation and palmitoylation motifs, which suggests that this region can be involved in protein stability (Figure 2G). The analysis of the nuclease activity of untagged and CFP-fused CAN2 revealed that mutants lacking the N-SNc, C-SNc domains and ABC protein-like motifs, are completely inactive (Figure 2H). Instead, deletion of the amino acid sequence showing a similarity to the anti-codon binding domain decreases but does not eliminate CAN2 DNase activity. This is particularly interesting, given the fact that this region is located inside the catalytic domain, but apparently is not crucial for CAN1/2 DNase and RNase activities (Figure 2C).

### The expression of CAN1 nuclease is induced during xylogenesis and leaf senescence and correlates with host-pathogen interactions, whereas the expression of CAN2 is generally constitutive

In order to establish gene expression profiles for both CAN nucleases we analyzed microarray experiments reported by Genevestigator [29]. This Database has collected results of 419 microarray experiments (7137 samples) performed by authors exploring various aspects of *Arabidopsis* biology. Since in many cases the same determinant of gene expression was analyzed independently by several research groups, in our opinion their results provide an opportunity to obtain verifiable and objective information about the expression patterns of selected genes.

**Figure 3:**
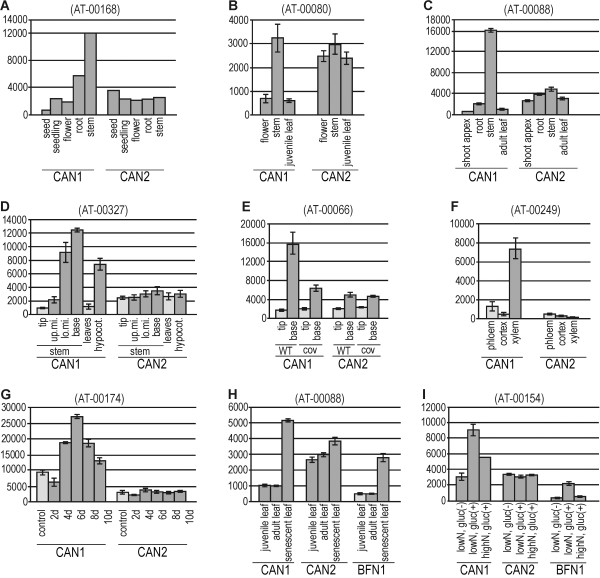
**Microarray-based expression analysis of CAN1 and CAN2 genes at different developmental stages of *Arabidopsis thaliana. ***Microarray expression data were retrieved from the Genevestigator database and processed as described in the Methods section. Numbers in brackets refer to the experiment ID from Genevestigator. Expression levels of genes encoding CAN1 [TAIR:At3g56170] and CAN2 [TAIR:At2g40410] proteins are displayed as signal values (y-axis) calculated for both genes in a given experiment. The x-axis indicates treatment conditions as described in the text and below. Light gray bars – controls, dark gray bars – expression in response to treatments within a given experiment. (**A-C**) Three examples of experiments demonstrating predominant expression of the CAN1 gene in *Arabidopsis* stem. (**D-E**) Two experiments showing the dependence of CAN1 expression on the stem growth phase. The abbreviations shown on the x-axis indicate the parts of stem: (tip) – stem tip, (up.mi.) - upper middle part of stem, (lo.mi.)- lower middle part of stem, (base) - stem base. (cov) - *A. thaliana cov* mutant affecting the vascular tissue development. (**F-G**) Correlation of CAN1 expression with xylem development in the root vascular bundle (**F**) and in cells induced to differentiate into tracheary elements (**G**). The abbreviations (2d-10d) indicate the number of days after culture induction. (**H-I**) correlation of CAN1 expression level with age-dependent (H) and sugar treatment induced senescence (I). The abbreviations (low/high N) and (gluc +/−) indicate the nitrogen and glucose concentration, respectively, in the culture medium. Expression profiles of senescence-associated BFN1 nuclease are also shown.

We began our studies by examining CAN1 [TAIR:At3g56170] and CAN2 [TAIR:At2g40410] genes expression during different developmental processes. In principle all microarray experiments conducted to compare the *Arabidopsis* tissue specific transcription profiles clearly indicate that CAN1 nuclease is preferentially expressed in stems and roots (Figure 3A-C, Additional file 1). On the other hand, low expression of CAN1 mRNA was observed in seeds, seedling, young leaves and flowers. In contrast to CAN1, the organ specific variability of CAN2 expression was relatively small. The analysis of other microarrays revealed that transcription of CAN1 is low in the upper part of the stem but significantly increased in the lower part (Figure 3D, E). Since these parts of the stem differ mainly in the degree of xylem development, this suggests that CAN1 nuclease can be involved in this process. This assumption has been confirmed by experiments conducted to identify the genes involved in development of different parts of the vascular bundle, i.e. xylem, phloem, cortex, (Figure 3F), as well as genes activated in cells induced to transdifferentiate into xylem tracheary elements (Figure 3G).

The level of CAN1 expression in young and mature leaves is low but it increases with leaf aging. This effect was observed in developmental leaf senescence (Figure 3H) as well as in leaves that have been induced to senesce by nitrogen deficiency (Figure 3I). Figure 3H-I shows that the expression pattern of CAN1 nuclease in senescent leaves is analogous to the AT1G11190 gene encoding the well defined BFN1 nuclease involved in leaf senescence [5,6]. Both genes, AT1G11190 (BFN1) and At3g56170 (CAN1), have also been included in the list of about 800 genes showing at least a 3 fold up regulation during leaf senescence, identified by Buchanan-Wollaston *et al.*[30].

**Figure 4:**
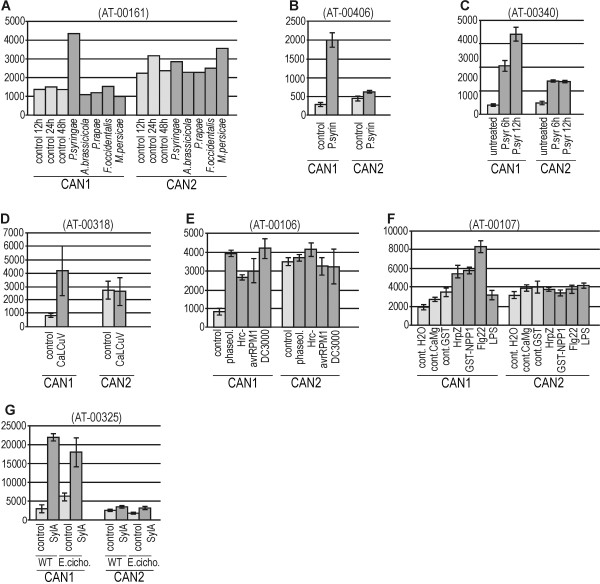
**Microarray-based expression analysis of CAN1 and CAN2 genes in response to various stimuli associated with pathogenesis.** Microarray expression data were retrieved from the Genevestigator database and processed as described in the Methods section. Numbers in brackets refer to the experiment ID from Genevestigator. Expression levels of genes encoding CAN1 [TAIR:At3g56170] and CAN2 [TAIR:At2g40410] proteins are displayed as signal values (y-axis) calculated for both genes in a given experiment. The x-axis indicates treatment conditions as described in the text and below. Light gray bars – controls, dark gray bars – expression in response to treatments within a given experiment. Detailed references to individual experiments are given in Additional file 2. (**A**) Microarray study showing specific activation of CAN1 expression in response to bacterial infection. Plant responses to the following microbial and insect pathogens are presented: leaf bacterium (*Pseudomonas syringae* pv. tomato), pathogenic leaf fungus (*Alternaria brassicicola*), tissue-chewing caterpillars (*Pieris rapae*), cell-content-feeding thrips (*Frankliniella occidentalis*) and phloem-feeding aphids (*Myzus persicae*). (**B-D**) Three experiments showing the effect of *P. syringae* (B-C) and DNA virus (cabbage leaf curl virus-CaLCuV) (D) on CAN1 gene expression. (**E**) Influence of different *P. syringae* strains on CAN1 expression. (**F**) Influence of different pathogen-derived elicitors on CAN1 gene expression. (HrpZ) - Harpin elicitor, (NPP1) - necrosis-inducing *Phytophthora* protein 1, (Flg22) – flagellin, (LPS) – lipopolysaccharide. (**G**) Expression of CAN1 gene in plants treated with Syringolin A (SylA) alone and with a combination of Syringolin A and *Erysiphe cichoracearum* infection.

Numerous microarray studies have demonstrated upregulation of CAN1 expression during environmental stresses. Similar to developmental processes, external stimuli significantly modulate expression of CAN1 (At3g56170) but weakly affect expression of CAN2 gene (At2g40410). We observed that among all the tested factors those that are related with pathogenesis have the strongest influence on CAN1 expression. The analysis of the experiments presented in Figure 4A-D and Additional file 2 clearly indicates that upregulation of this gene is stimulated by some bacteria and viruses, while fungi, nematodes and insects do not exert such an effect. Among the ten experiments conducted to study various aspects of *Pseudomonas* sp. infection, nine revealed an elevated level of CAN1 mRNA in relation to controls in tissues attacked by this pathogen (Figure 4A-C, Additional file 2). The transcription of the CAN1 gene was also stimulated by *E.coli* (Additional file 2, AT-00202) and dsDNA virus, CaLCuV (Figure 4D). Other experiments have shown that activation of CAN1 does not depend on the virulence of the *Pseudomonas* strain (vir/avir) (Figure 4E) and also occurs after elicitor treatment. Three of four available experiments investigating the influence of flagellin on gene expression have shown that this elicitor can activate CAN1 transcription (Figure 4F, Additional file 2). The particularly strong upregulation of the CAN1 gene was observed in two experiments studying the influence of syringolin A, the virulence factor secreted by *P.syringae* (Figure 4G, Additional file 2).

**Figure 5:**
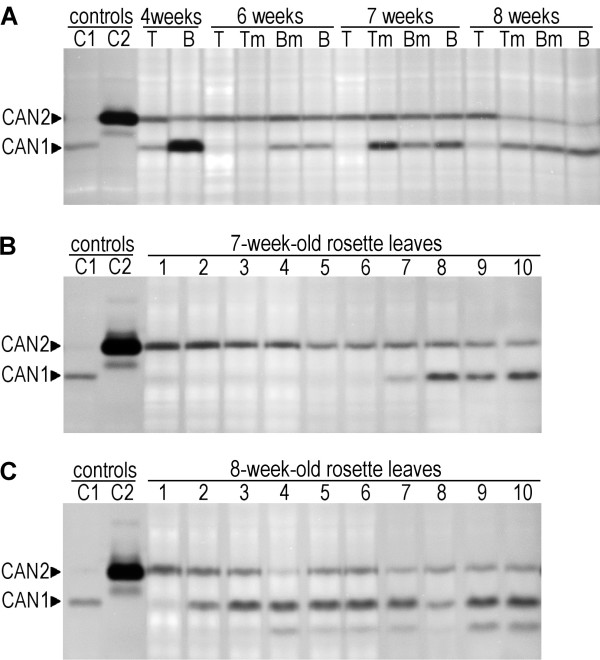
**Endogenous CAN1 and CAN2 activity.** (**A**) Changes in endogenous CAN1 and CAN2 activity during stem development. DNase activity of protein extracts from the 4–8 week old stems were analyzed. The abbreviations for the gel tracks refer to the part of stem: (T) – stem tip, (Tm) - upper middle part of stem, (Bm) - lower middle part of stem, (B) - stem base. CAN1 (C1) and CAN2 (C1) nucleases transiently expressed in protoplasts were used as positive controls. (**B-C**) The endogenous CAN1 and CAN2 nuclease activity of individual leaves of 7 week old (**B**) and 8 week old (**C**) rosettes. Consecutive leaf extracts are arranged from the youngest (1) to oldest (10) leaf of rosettes. The CAN1 (C1) and CAN2 (C1) controls as above (Figure 5A).

To verify the data obtained from the microarray experiments we examined whether suggested mRNA expression profiles of CAN1 and CAN2 genes correspond to any endogenous nucleolytic activity. We analyzed nuclease activity profiles of developing stem and senescing leaves. The development of xylem begins in the lower part of the stem and this zone successively moves towards the upper part of the growing stem. According to the scheme presented by Brown *et al.*[31] we analyzed the protein extracts isolated from different parts of stems obtained from plants aged from 4 to 8 weeks. As shown in Figure 5A, in-gel nuclease activity assay revealed that the samples prepared from stems contain two nucleases migrating to the same position as over-expressed CAN1 and CAN2 used as a control. The activity of endogenous nucleases corresponding to CAN2 was essentially constant, which agrees with the observation derived from the microarray data. In contrast, the activity of the endogenous nuclease migrating in an identical fashion as CAN1 is much more variable, i.e. it is invisible or weak in the upper part of stems and increases towards the stem base.

To identify the nucleases associated with senescence we analyzed protein extracts obtained from the individual rosette leaves of 7 and 8 week old plants. As presented in Figure 5B and C, endogenous nuclease activity corresponding to CAN1 appears for the first time in older leaves of a 7 week old rosette and becomes more common in the 8 week old rosette leaves. Thus, this result supports the role of CAN1 nuclease in age-dependent development. The activity profile of CAN2 nuclease identified in 7 and 8 week old leaves as well as in the leaves of younger plants (data not shown) was much more constant, which supports the hypothesis that this nuclease is characterized by a constitutive expression.

The assumption that observed endogenous nuclease activities refer to CAN1 and CAN2 proteins stems from the facts that corresponding proteins possess exactly the same electrophoretic mobility, catalytic requirements, and unusual resistance to heat denaturation, detergents and 2-mercaptoethanol. Resistance to a reducing agent provides an opportunity to distinguish endogenous plant staphylococcal-like nucleases from plant S1 type nucleases, which possess disulphide bonds and in our experience are inhibited by 2-mercapthoethanol (data not shown). This allows us to conclude that microarray mRNA expression data, confirmed by our protein activity assays, together suggest that expression of CAN1 nuclease is associated with different forms of PCD, such as xylem development or senescence, while CAN2 expression is generally constitutive.

### The CAN1 and CAN2 nucleases are anchored to the plasma membrane, probably via N-terminal myristoylation and palmitoylation

**Figure 6:**
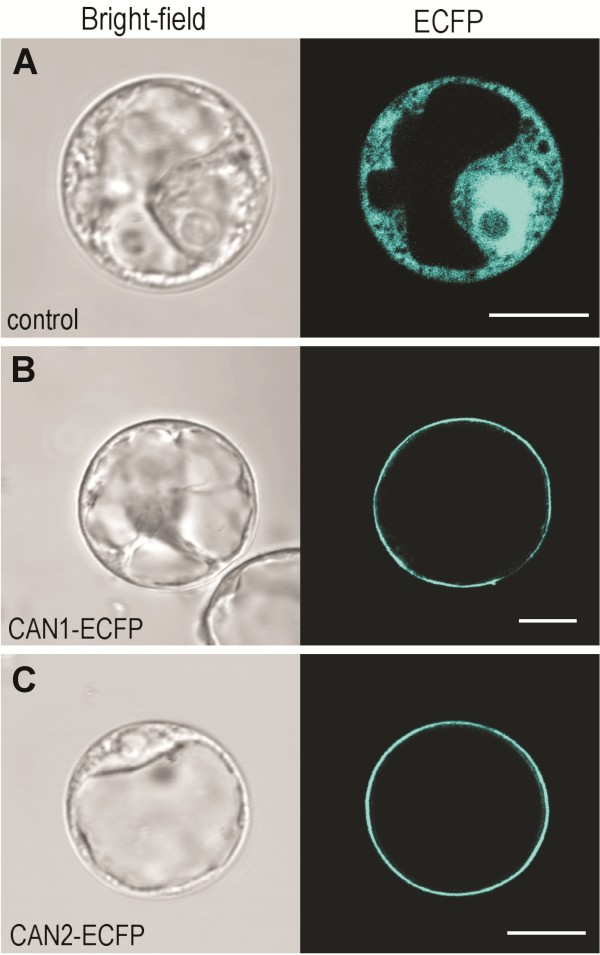
**Plasma membrane localization of CAN1 and CAN2 nucleases in protoplasts.** ECFP alone and ECFP-fusion proteins were transiently expressed in *Arabidopsis* root cell protoplasts. Localization was analyzed by confocal laser scanning microscopy (right half of each panel). The corresponding bright-field images are shown on the left halves of panels. (**A**) Protoplast transformed with empty vector (pSAT6A-ECFP) as a control shows characteristic cytoplasmatic and nuclear localization (**B**) Expression of the CAN1-ECFP fusion construct. (**C**) Expression of the CAN2-ECFP fusion construct. Clear plasma membrane localization of both nucleases is seen. The length of the white scale bar at the right bottom of each panel corresponds to 10 μm.

**Figure 7:**
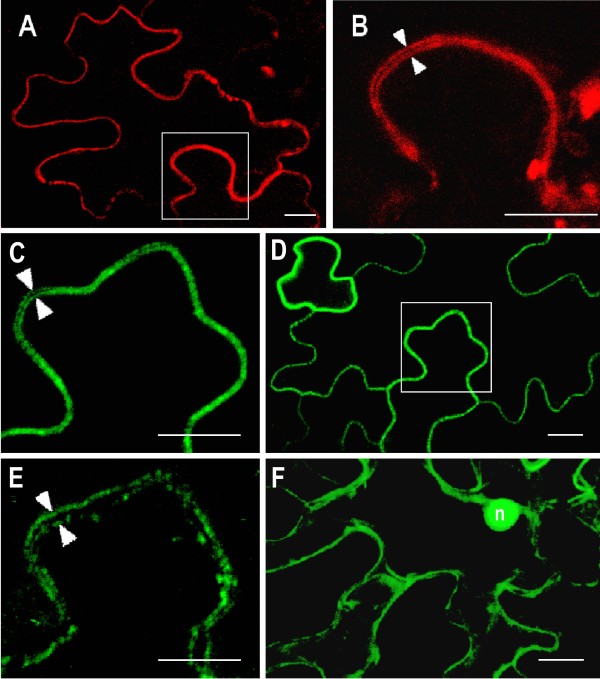
**Plasma membrane localization of CAN1-RFP and CAN2-GFP nucleases transiently expressed in *Arabidopsis *leaf epidermal cells. **Confocal images were taken two days after *A. tumefaciens* infiltration. (**A**) An epidermal cell expressing CAN1-RFP fusion protein. (**B**) Higher magnification image of the box in (A). White arrowheads indicate plasma membranes of adjacent cells. (**C-E**) Epidermal cells expressing the CAN2-GFP fusion protein. (**C and E**) Higher magnification images of the box in (D) show plasma membranes of adjacent cells before (**C**) and after (**D**) plasmolysis. (**F**) Epidermal cells transformed with empty vector pSITE-2NB-GFP as a control. Strong signal around the nucleus (n) and numerous cytoplasmic strands show characteristic cytoplasmatic localization of GFP protein. The length of the white scale bar at the right bottom of each panel corresponds to 10 μm.

In order to determine the cellular distribution of CAN nucleases, the translational fusion constructs, CAN1-ECFP and CAN2-ECFP, were transiently expressed in *Arabidopsis* root protoplasts. As a negative control, an empty vector plasmid, pSAT4A-ECFP (expressing ECFP alone), was used. As demonstrated in Figure 6A, ECFP alone, used as a control, is typically distributed throughout the cytoplasm and nucleus. In turn, intensive and sharp fluorescent rings were visible around the protoplasts with over-expressed CAN1 and CAN2 nucleases fused to ECFP (Figure 6B, C). The same subcellular location of both proteins we observed in transformed leaf protoplasts (Additional file 3). Protoplasts transfected with the same constructs but analyzed via fluorescence microscopy displayed uniform fluorescence of the protoplast surfaces (Additional file 4). These fluorescence images suggest that both nucleases are associated with plasma membrane. To ensure that this unusual location of proteins possessing nuclease activity is not the artificial result of the protoplast preparation procedure we also analyzed the location of both nucleases in plant cells transformed via *Agrobacterium tumefaciens*. As shown in Figure 7A and D, the fluorescence of CAN1-RFP and CAN2-GFP fusion proteins appeared as sharp lines along the edges of transformed epidermal cells. Magnifications of selected areas showed that the fluorescence signal forms two parallel lines indicating plasma membranes of two neighboring cells (Figure 7B, C). Moreover, as presented in Figure 7E, drought induced plasmolysis caused displacement of the fluorescence signal towards the cell center, which further proves the association of CAN nucleases with the plasma membrane.

**Figure 8:**
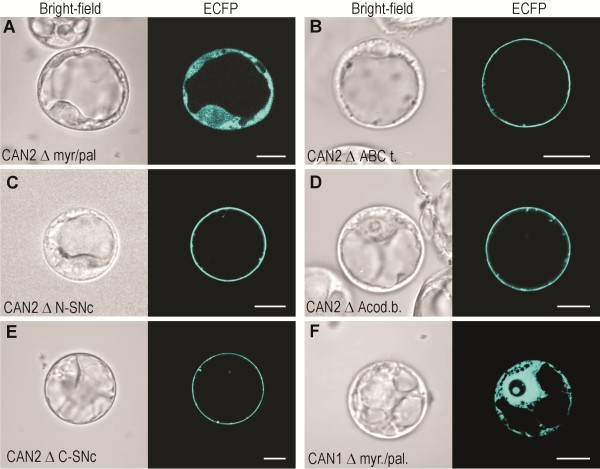
**Subcellular localization of CAN nucleases deletion mutants.** ECFP-fusion proteins were transiently expressed in * Arabidopsis * root cell protoplasts. The bright-field images are shown in the left part of each panel and corresponding confocal laser scanning images are on the right. The images show the cellular location of the ECFP fused proteins lacking the following consensus sequences: (**A**) CAN2 mutant lacking N-myristoylation and palmitoylation consensus sequences. (**B**) CAN2 mutant lacking the ABC transporter-like sequence. (**C**) CAN2 mutant lacking the N-SNc domain. (**D**) CAN2 mutant lacking the anticodon binding-like sequence (**E**) CAN2 mutant lacking the C-SNc domain. (**F**) CAN1 mutant lacking N-myristoylation and palmitoylation consensus sequences. The abbreviations of mutated domains as in Figure 2F The length of the white scale bar at the right bottom of each panel corresponds to 10 μm.

The bioinformatic analysis of CAN nucleases amino acid sequences did not reveal any pronounced hydrophobic regions that could represent transmembrane domains. To identify the segments potentially interacting with the plasma membrane we applied deletion mutants of the CAN2 nuclease (Figure 2F) for transfection experiments. As shown in Figure 8B-E, the deletion mutations created in the ABC-like domain, the anticodon binding-like domain and both fragments of SNc domains did not affect the plasma membrane localization of CAN2. Insted, deletion of the N-end fragment containing the myristoylation/palmitylation motifs significantly affected subcellular localization of this nuclease (Figure 8A). The removal of the myristoylation/palmitylation motifs from the CAN1 nuclease resulted in the same effect (Figure 8F). Since it is known that N-terminus myristoylation is required for membrane targeting and that palmitoylation of proximal cysteine residue greatly stabilizes membrane association, we concluded that CAN nucleases are most probably anchored to the plasma membrane via these posttranslational modifications.

We took into account that CAN nucleases could change their location in response to various cellular and exogenous factors related to PCD. To verify such a possibility the protoplasts containing over-expressed CAN1-ECFP and CAN2-ECFP were then subjected to treatment with the following factors, in the ranges given in brackets: H_2_O_2_ (0.1-1mM), heat stress (36-55°C), salt stress (NaCl 0.1-1M), flg22 peptide (0.1-1mM), *E. coli* strain DH5α (OD 0.01) and *A.tumefaciens* strain GV3101 (OD 0.01). Moreover, we compared the cellular location of CAN nucleases over-expressed in protoplast prepared from the cells of young (6 week old), mature (8 week old) and early senescent (12 week old) rosette leaves. However, we did not observe any influence of the above treatment on CAN subcellular location (data not shown). Moreover, we did not detect over-expressed proteins in the protoplast medium which suggests that under the tested conditions they are not secreted from the cell (data not shown). These results show that CAN nucleases probably do not change their cellular localization under stress conditions that could trigger PCD or plant defense response.

## Discussion

Despite the significant sequence similarity, eukaryotic proteins possessing SNase domains appear to fulfill different biochemical and biological functions. For example, the Tudor staphylococcal nuclease (Tudor-SN) possessing both DNase and RNase activities, specifically interacts with and promotes the cleavage of dsRNA substrates. Since this protein is a component of the RISC complex, it is expected that it contributes to the RNA degradation observed in RNAi [32]. The other eukaryotic homologue of staphylococcal nuclease, the parB, acquired a novel function during evolution, acting as a redox enzyme [33]. Our attempt to detect the potential nuclease activity of the *Arabidopsis* parB-like protein [TAIR:AT1G31170] which we performed in parallel to the study described in this paper also did not reveal any nuclease activity of this protein (data not shown).

In this paper, we examined the molecular activity and biological function of enzymes belonging to the *Arabidopsis* two member protein family characterized by the presence of a single SNase domain. Transient expression assay using protoplasts, together with biochemical approaches, enabled us to show that CAN1 and CAN2 exhibit calcium dependent deoxyribonuclease activities similar to that of the bacterial SNases. We found that, despite the presence of conserved calcium binding and catalytic amino acid residues in CAN SNase domains, they also contain an additional, conserved motif showing strong homology to the anticodon binding domain of some bacterial tRNA synthetases. Although both of these domains appear to be of bacterial origin, they do not occur together in such an arrangement in any known prokaryotic protein. How the putative RNA binding motif affects DNase activity of plant nucleases remains unknown. However, because the deletion of this motif decreases, but does not eliminate DNase and RNase activities, it could be speculated that the anticodon-binding like domain contributes to the enhancement of the CAN nucleases catalytic activity.

Despite their amino acid sequence similarity, CAN1 and CAN2 exhibit some significant differences. We demonstrate that, while CAN1 nuclease digests the single stranded and double stranded DNA with comparable efficiency, CAN2 strongly prefers single-stranded DNA as its substrate. Moreover, the genes encoding both nucleases exhibited fundamentally different expression profiles which may suggest that, despite having the same subcellular distribution, they do not cooperate in a common biological process.

It seems that the most remarkable feature of both CAN nucleases is their unusual cellular localization. Nucleases that have been studied so far were identified in cellular compartments containing nucleic acids, such as the nucleus [1], plastids and mitochondria [34] or in the organelles where they are stored before final application, for example, vacuole [12], lysosome [1] and other intracellular membrane vesicles [6]. To our knowledge, the CAN enzymes described in this paper are the only eukaryotic nucleases associated with the plasma membrane. Considering the potential function of CAN nucleases in the context of their unusual location, it might be tempting to speculate that plasma membrane nucleases could be involved in defense response against pathogen invading plant cells. This assumption could be supported by our finding that expression of CAN1 correlates well with bacterial and viral infection. However, overall analysis of CAN1 expression patterns displayed that activation of its gene is also associated with developmental processes unrelated to the pathogenic response, such as xylem formation and leaf senescence. Thus, it seems that the CAN1 nuclease is not involved in the pathogen’s genetic material degradation, unless it has more than one function. Similarly, it cannot currently be excluded that a second member of this family, constitutively expressed CAN2 nuclease, is involved in some, as yet uncharacterized mechanism of pathogen DNA processing at the cell surface or in the endosomes.

Detailed analysis of the CAN1 expression profile revealed that its gene is specifically up-regulated in two developmental processes leading to controlled cell death, i.e. xylogenesis and senescence. Induction of CAN1 expression in response to bacterial and viral attack may also be related to cell death, because recognition of avirulent pathogens can trigger resistance-associated PCD, a hypersensitive response, which also involves DNA degradation. It should be noted that some HR induced tobacco deoxyribonucleases [35], whose coding sequences have not yet been identified, possess catalytic properties similar to those of CAN1 and CAN2 as presented in this paper. The assumption that CAN nucleases can be involved in PCD was also proposed by Gu *et al.*[23], who observed the correlation of increased expression of their cucumber homologue, CsCaN, with primordial anther-specific DNA damage. Moreover, it is worth noting that ability of CAN nucleases to efficient and nonspecific degradation of DNA as well as RNA is also characteristic for PCD associated degradation processes.

It is believed that one of the main function of the degradation processes occurring during some types of plant PCD, mainly in the case of senescence, is redistribution of nutrients from dying cells to the expanding or sink tissues. Since nitrogen deficiency is the major nutritional factor limiting plant growth, most research has focused on understanding the reutilization of reduced nitrogen released from proteins, especially from Rubisco, which is the most abundant protein in plants. Available evidence shows that this is a multistage process characterized by intra- or intercellular compartmentalization [36]. Degradation of Rubisco initiated by plastidial and vacuolar endopeptidases is continued into single amino acids and then to ammonium, which is finally reassimilated into glutamate, glutamine and aspartate. Since the concentrations of these amino acids in phloem sap increase during late senescence, it is believed that they are the major forms of long-distance translocatable products of protein degradation [37]. Although the initial proteolysis occurs in the senescent mesophill cells, subsequent steps, catalyzed by glutamate dehydrogenase (GDH) or glutamine synthetase (GS), occur in cells adjacent to vascular bundles, such as vascular parenchyma or companion cells [38].

The catabolic pathways responsible for the recycling of nucleic acid-derived nutrients are much less understood than those concerning proteins. Nucleotides are the second main source of cellular nitrogen as well as an important reservoir of phosphorus. However, it is still unknown to what extent nucleic acids are degraded during PCD, and how these degradation products are processed for long-distance transport via the phloem. Nucleic acid degradation seems to be initiated by S1-type enzymes, such as BFN1, Zen1, Ben1, whose expression correlates with different types of plant PCD. The nature of the reaction products generated by these nucleases is still not clear but, since these enzymes possess endonucleolytic activity, they probably produce DNA fragments of various sizes. However, the study of phloem sap content during senescence did not reveal any DNA fragments or single deoxynucleotides. Instead, pronounced expression of some nucleobase and phosphorus transporters was demonstrated in phloem vessels [39,40] suggesting that some additional reaction(s) must lead to further degradation of the S1 type nuclease derived DNA fragments and subsequent nucleotide catabolism. Moreover, assuming that nucleotides, similar to amino acids, are catabolized in cells associated with vascular bundles, some mechanism responsible for intercellular transfer of DNA degradation products from dying cells should also be considered. This, as yet hypothetical, process would be in analogy with those, that enable the mycoplasma to acquire nucleic acid precursors. Since the CAN1 nuclease presented here seems to be associated with PCD and its properties resemble the mycoplasma membrane nucleases, in the future we intend to verify the hypothesis that this enzyme combines DNA degradation with transfer of degradation products between the dying cells and the cells that are responsible for further nucleotide catabolic processes.

The current state of knowledge does not allow us to clearly explain at what stage of above described processes the CAN nucleases may be involved and various hypotheses concerning their function(s) require further studies. However, the identification of a novel class of nucleases characterized by their plasma membrane location indicates that plant cells possess additional, so far uncharacterized, mechanisms responsible for DNA degradation.

## Conclusions

One of the main function of plasma membrane is to provide a highly selective transport of molecules important to cell growth. An intensive export of nutrients released from degraded macromolecules is observed in plant tissues undergoing PCD. Although nucleic acids are a rich source of nitrogen, phosphorus and nucleotide bases little is known about the export of DNA and RNA degraded products from dying cells. Nucleases responsible for genomic DNA degradation during PCD were identified in cellular compartments containing nucleic acids or in the organelles where they are stored before final application. In this paper we present evidence showing that two plant staphylococcal-like nucleases belong to a new, as yet unidentified class of eukaryotic nucleases, characterized by unique plasma membrane localization. The identification of this class of nucleases indicates that plant cells possess additional, so far uncharacterized, mechanisms responsible for nucleic acid degradation.

## Methods

### Bioinformatic analysis

The cDNA sequences encoding *Arabidopsis* CAN1 [TAIR:At3g56170], CAN2 [TAIR:At2g40410] and bacterial proteins presented in Figure 1 [NCBI:YP_004267097, YP_004174706, YP_004267097; GenBank: EGW73615, EGW73615) were obtained from NCBI ( http://www.ncbi.nlm.nih.gov). For the alignment of the amino acid sequences, the BLASTP ( http://blast.ncbi.nlm.nih.gov/Blast.cgi) and CLUSTALW ( http://www.ebi.ac.uk/Tools/clustalw2) programs were employed. Conserved protein domains and domain architecture were identified using online tools ( http://www.ncbi.nlm.nih.gov/Structure/cdd/wrpsb.cgi).

The Myristoylator ( http://www.expasy.ch/tools/myristoylator/) program and CSS-Palm software ( http://csspalm.biocuckoo.org/index.php) were used to predict N- terminal myristoylation and palmitoylation sites, respectively. Prediction of transmembrane regions was performed using the TMHMM ( http://www.cbs.dtu.dk/services/TMHMM-2.0), SOSUI ( http://bp.nuap.nagoya-u.ac.jp/sosui/sosui_submit.html) and PSORT programs ( http://www.psort.org/).

In silico analyses of gene expression patterns were performed with publicly available microarray expression data provided by Genevestigator [29] ( https://www.genevestigator.ethz.ch/at). The Meta-profile-Stimulus tool of the Genevestigator software was used to estimate the levels of gene expression in response to different external stimuli. The signal values of individual arrays were obtained from the Meta-profile Northern tool plots and transferred to a Microsoft Excel spreadsheet for the calculation of the standard error values and to create charts.

### cDNA cloning and expression

Total RNA was isolated from 30-day-old *Arabidopsis thaliana* rosette leaves using TRIzol reagent (Invitrogen) according to the manufacturer's instructions. The mRNA was reverse-transcribed into single-stranded cDNA using an oligo (dT) primer and MMLV reverse transcriptase (Promega) according to the protocol supplied along with the enzyme. The nested PCR was used to amplify cDNA encoding CAN1 and CAN2 proteins. The first round of amplification was performed with specific pairs of primers designed for both cDNAs using the Primer3 program ( http://biotools.umassmed.edu/bioapps/primer3_www.cgi). The PCR products were used as a template for the second PCR reaction performed with primers that contained artificial SalI and BglII restriction endonuclease sites at their 5' and 3’ ends, respectively. The primers used for RT-PCR amplification are listed in Table 1. An expression vector designed to express HA-tagged fusion proteins was prepared by ligation of two annealed, complementary oligonucleotides encoding the HA-tag domain with pSAT6A vector [41]. The PCR products encoding CAN1 and CAN2 proteins with stop codons were digested with SalI and BglII restriction endonucleases and ligated into pSAT6A vector. The PCR products lacking the stop codon were digested with the same pair of restriction enzymes and ligated in frame with 5’end of HA domain or ECFP reporter gene of pSAT6A-HA and pSAT6A-ECFP-N1 vectors, respectively. The resulting constructs were confirmed by sequencing.

**Table 1:** **Primers used for PCR and RT-PCR amplification of *Arabidopsis *CAN1 and CAN2 cDNAs**

**primer name**	**sequence**
CAN1For	5’AAATGGGTAACGCGATTAGG3’
CAN1Rev	5’CATTAATTGCCTCCACGTTTG3’
CAN2For	5’TGGGTGAAAGATGGGTAACG3’
CAN2Rev	5’CCCTCAACAATCACAAATCTCA3’
CAN1Sal	5’ACCGTCGACATGGGTAACGCGATTAGGTTA3’
CAN1Bgl	5’GGGAGATCTGATTGCCTCCACGTTTGTTCT3’
CAN2Sal	5’GTTGTCGACATGGGTAACGCTCTTACGTTT3’
CAN2Bgl	5’CCCAGATCTGTTCCCGCCTATTATTCTTTC3’

To transform *Arabidopsis* leaf epidermal cells, vectors from the pSITE series [42] were used. PCR products encoding CAN1 and CAN2 amino acid sequences lacking their native stop codons and flanked by attB1 and attB2 sites were cloned into pDONR donor vectors according to the manufacturer's protocols (Invitrogen). Then, the CAN1 and CAN2 coding sequences were transferred by Gateway recombination reactions into the pSITE-4NB (RFP) and pSITE-2NB (GFP), respectively. The destination vectors were transformed into *Agrobacterium tumefaciens* GV3101 by the conventional freezing-and-melting method.

### SDS-PAGE and the Western blot

SDS-PAGE was performed according to standard protocols with the Hoefer Mighty Small II gel system. Proteins were transferred to Amersham Hybond-P PVDV membrane with the Biometra Fastblot B43 semi-dry transfer system (0.8 mA/cm^2^, 1 h). The membrane was blocked and washed according to the manufacturer’s instructions. The primary anti-HA antibody (Santa Cruz Biotechnology) and secondary anti-rat HRP-conjugated antibody (Sigma) were diluted 1:200 and 1:5000, respectively. Immunological detection of proteins was performed with the GE Healthcare ECL Plus Western Blotting Detection System.

### Transient gene expression in *Arabidopsis thaliana* leaves and protoplasts

Protoplasts from *Arabidopsis* root cells (kindly provided by prof. Elizabeth Jamet) and from leaf mesophyll were prepared as described by He *et al.*[43] and Yoo *et al.*[44], respectively. Twelve hours after PEG-mediated transformation, the protoplasts were either prepared for microscopic analysis or harvested and used to prepare the protein extracts for *in vitro* assays. Protein extracts were prepared by direct protoplast lysis in protein sample buffer (30% glycerol (v/v), 160 mM Tris-Cl pH 6.8, 6% SDS) supplemented with 5 mM EDTA and 4% (vol/vol) 2-mercaptoethanol. An aliquot of each extract was taken to measure the protein concentration using Bradford reagent (Pierce). The remainder of the extracts was then frozen rapidly at −20°C.

For transient expression in *Arabidopsis* leaves, the *Agrobacterium* strains harboring the destination vectors were diluted in transformation buffer (10mM Mes, pH 5.8; 10mM MgCl_2_; 0.15mM acetosyringone) to an OD_600_ of 0.05 and applied with a syringe to the underside of the leaves of 4–6 week-old plants.

### In-gel nuclease activity assay

The detection of DNase activities was performed as described previously [9] with the following modifications. The stacking gel was enriched with 1 mM EDTA to remove the residual ions present in protein extracts. The resolving minigel contained sonicated calf thymus DNA (0.008 mg/ml). For single-stranded DNase activity, DNA was boiled for 5 min prior to pouring the gel. To identify both the activity of transiently expressed nucleases and endogenous nuclease activity 1μg and 10μg of protein extracts were used, respectively. Protein extracts were incubated for 5 min at 100°C in standard sample buffer enriched with 2-mercaptoethanol. Electrophoresis was performed at +4°C applying 10 V/cm. After electrophoresis, the resolving gels were soaked twice for 20 min at room temperature in 20% (v/v) isopropanol. Subsequently, the gels were washed twice for 15 min and incubated for 48 hours in renaturation buffer containing 1% (v/v) Triton X100 and 20 mM Tris-Cl (pH 8.0). To test the influence of low pH on nuclease activity, 25 mM sodium acetate (pH 5.5) was used instead of Tris-Cl. Depending on the experiment, the renaturation buffers were supplemented with either 1-10mM CaCl_2_, 0.2 mM ZnCl_2_ or 5mM EDTA. After incubation, the gels were washed and then stained in ice cold buffer containing 10 mM Tris, 1mM EDTA and 0.01 mg/ml ethidium bromide to reveal the position of nucleases.

### Preparation of protein extracts from *Arabidopsis* stems and leaves

*Arabidopsis thaliana* plants (Columbia ecotype) were grown in sterile soil pots (50 ml) in the phytotron (16 h light, 8 h dark). Day and night temperatures were set at 22°C and 18°C, respectively. Plants were watered and fertilized daily with MS mineral solution. Selected organs were harvested at various stages of growth as indicated in the Results section and used directly to prepare protein extracts. The plant pieces were placed into liquid nitrogen for 5 min. After thawing, 0.05-0.2ml sample buffer (30% glycerol (v/v), 160 mM Tris-Cl pH 6.8, 6% SDS) supplemented with 5 mM EDTA and 4% (vol/vol) 2-mercaptoethanol was poured over the samples which were then shaken (1400 rpm) for 5 min. at 99°C. Finally, following centrifugation at 3000×g for 5 min, the protein extracts were assayed for protein concentration and stored at −20°C.

### Confocal Microscopy Analysis

Confocal laser scanning microscopy was performed using a NIKON A1Rsi inverted confocal microscope. To image fluorescent protein fusions in *Arabidopsis thaliana* protoplasts a PL APO100X OI (NA = 1.4) objective was used. ECFP fluorescence was excited by the 457 nm argon laser and detected using a custom 482/35 nm band-pass emission filter. To visualize protein fusions in leaf epidermis a CFI LWD APO 40X WI (NA = 1.15) objective was used. eGFP fluorescence was excited using the 488 nm argon laser and detected using a custom 525/50 nm band-pass emission filter. mRFP fluorescence was excited using a 561nm helium-neon laser and detected using a custom 595/50 nm band-pass emission filter. Data were collected as single optical sections or as a series of Z-stacks and processed using ImageJ software.

## Abbreviations

CAN: Calcium dependent nuclease; PCD: Programmed cell death; SNc: Staphylococcal nuclease.

## Competing interests

The authors declare that they have no competing interests.

## Authors’ contributions

KL made a substantial contribution to the conception and design of research, carried out all molecular biology experiments, and drafted the manuscript. EP has made contribution to the conception of the study, interpretation of data and participated in drafting the manuscript. MS: carried out all microscopic analysis. NW and SS helped in plant protein extract preparation and evaluation. PW: participated in the coordination of the overall study. All authors read and approved the final manuscript.

## Supplementary Material

Additional file 1 **Microarray experiments extracted from the Genevestigator website, examining *Arabidopsis thaliana *developmental gene expression.** Click here for file

Additional file 2 **Microarray experiments extracted from the Genevestigator website, examining expression of *Arabidopsis *thaliana genes in response to various pathogen infections and elicitor treatments.** Click here for file

Additional file 3 Plasma membrane localization of CAN1 and CAN2 nucleases in leaf protoplasts. Click here for file

Additional file 4 The plasma membrane localization of CAN nucleases by fluorescence microscopy. Click here for file
