# Supplementary material for: Plant plasma membrane-bound staphylococcal-like DNases as a novel class of eukaryotic nucleases
Source: BMC Plant Biol. 2012 Oct 26;12:195. doi: 10.1186/1471-2229-12-195 (PMC3505149; doi:10.1186/1471-2229-12-195)
Supplement: Additional file 1 — Microarray experiments extracted from the Genevestigator website, examining Arabidopsis thaliana developmental gene expression. [file 1471-2229-12-195-S1.pdf]

Additional file 1. Microarray experiments extracted from the Genevestigator website, examining *Arabidopsis thaliana* developmental gene expression. Experiment ID marked with an asterisk (*) refers to those experiments showing a positive correlation between CAN1 gene expression and the developmental stages described in the text.

Experiment ID	Title	Authors
AT-00168 * (Figure 3A)	GSE680/GSE1051: Transcript Profiling of Arabidopsis Plant Life Cycle / Seed development in LEAFY COTYLEDON1 mutants	Le BH et al. / Goldberg RB
AT-00080 * (Figure 3B)	GSE607: Study of gene expression in expanding leaves, stem and flowers of Col-0 plants	E Osborne / C Somerville
AT-00087 *	AtGenExpress: Development baseline I	Weigel Lab
AT-00088 * (Figure 3C,H)	AtGenExpress: Development baseline II	Weigel Lab
AT-00327 * (Figure 3D)	E-MEXP-265: Transcription profiles from Arabidopsis stem, leaf and hypocotyl tissue	Zeef L et al. / Brown D
AT-00093 *	AtGenExpress: root & shoot development	Scheres Lab
AT-00122 *	Transcription profiling of Arabidopsis leaves, roots and whole plants grown in high or low phosphate conditions for different lengths of time.	J. Mission, MC Thibaud, S Somerville
AT-00191 *	GSE5749: A gene expression map of the Arabidopsis root	Birnbaum K et al./ Schildknecht B
AT-00066 * (Figure 3E)	E-GEOD-5729: Transcription profiling of Arabidopsis continuous vascular (cov-1, cov-2) mutants	Turner S
AT-00249 * (Figure 3F)	GSE16468: Transcriptional and posttranscriptional regulation of transcription factor expression in Arabidopsis roots	Lee J et al. / Benfey P
AT-00174 * (Figure 3G)	In vitro tracheary element transdifferentiation of Col-0 suspension cells	Fukuda H et al. / Demura T
AT-00154 * (Figure 3I)	Expression patterns of genes induced by sugar accumulation during early leaf senescence	Nathalie Pourtau / Astrid Wingler
