# Supplementary material for: Plant plasma membrane-bound staphylococcal-like DNases as a novel class of eukaryotic nucleases
Source: BMC Plant Biol. 2012 Oct 26;12:195. doi: 10.1186/1471-2229-12-195 (PMC3505149; doi:10.1186/1471-2229-12-195)
Supplement: Additional file 2 — Microarray experiments extracted from the Genevestigator website, examining expression of Arabidopsis thaliana genes in response to various pathogen infections and elicitor treatments. [file 1471-2229-12-195-S2.pdf]

Additional file 2. Microarray experiments extracted from the Genevestigator website, examining expression of *Arabidopsis thaliana* genes in response to various pathogen infections and elicitor treatments. Experiment ID marked with an asterisk (*) refers to those experiments showing up regulation of the CAN1 gene in response to the respective treatment.

	Experiment ID	Title	Authors
bacteria	AT-00161 * (Figure 4A)	GSE5525: Transcriptome changes of Arabidopsis during pathogen and insect attack	Pieterse C et al. / Schildknecht B
	AT-00406 * (Figure 4B)	GSE18978: Arabidopsis thaliana mutant leaves treated with Pseudomonas syringae ES4326	Mitra R
	AT-00340 * (Figure 4C)	E-MEXP-1094: Expression profiles of Col-0 and transgenic lines overexpressing AtFAAH(At5g64440) after inoculated with nonhost pathogen Pseudomonas syringae pv. syringae at 0, 6 and 12 hours	Tang Y
	AT-00106 * (Figure 4E)	AtGenExpress: response to virulent, avirulent, typeIII-secretion system deficient and non-host bacterial pathogens	Kemmerling B / Nürnberger T
	AT-00204 *	GSE6556: Expression profiling of A. thaliana wild type Columbia-0 and mutant gh3.5-1D in response to pathogen Pst DC3000(avrRpt2)	Zhang Z et al. / He Z
	AT-00211 *	E-MEXP-546: Transcription profiling leading to the identification of novel components in the EDS1/PAD4-regulated defence pathwayabidopsis-Pst-eds1-pad4	Bartsch M / Gobbato E / Parker J
	AT-00202 *	GSE5520: Genome-wide transcriptional analysis of the compatible A. thaliana-P. syringae pv. tomato DC3000 interaction	Underwood W et al. / Schildknecht B
	AT-00391	GSE17464: Arabidopsis mutants treated with flg22, Alternaria brassicicola, Methyl jasmonate and Pst DC3000 bacteria	Bari R et al. / Jones J
	AT-00393 *	GSE19255: Microarray analysis of fdh1-3(avrB)	Yun B
	AT-00148 *	AtGenExpress: Pseudomonas half leaf injection	Xinnian Dong
viruses	AT-00318 * (Figure 4D)	E-ATMX-34: Effect of geminivirus Cabbage leaf curl virus on Arabidopsis Col-0 at 12 days post-inoculation during short day conditions	Ascencio-Ibanez T
	AT-00324	E-MEXP-509: Potyvirus turnip mosaic virus infection	Yang C
fungi	AT-00161. (Figure 4A)	GSE5525: Transcriptome changes of Arabidopsis during pathogen and insect attack	Pieterse C et al. / Schildknecht B
	AT-00023	GSE10323: Testing Arabidopsis for the presence of arbuscular mycorrhizal signalling pathways	Yap H et al. / Schildknecht B
	AT-00085	GSE431: pmr4 vs. wild-type	Nishimura MT et al. / Somerville S
	AT-00146	GSE5686: AtGenExpress: Pathogen Series: Response to Erysiphe orontii infection	Ausubel F et AL / Schildknecht B
	AT-00147	GSE5684: AtGenExpress: Pathogen Series: Response to Botrytis cinerea infection	Denoux C et al. / Schildknecht B
	AT-00309	GSE12856: Penetration resistance: Wildtype and ataf1-1 mutant response to Bgh 12 h after inoculation, 2*2 factorial design	Jensen MK
	AT-00391	GSE17464: Arabidopsis mutants treated with flg22, Alternaria brassicicola, Methyl jasmonate and Pst DC3000 bacteria	Bari R et al. / Jones J
	AT-00453	GSE26679: comparison of powdery mildew-induced gene expression between Col-0 and the edr1 mutant	Innes RW / MChristiansen K
oomycete	AT-00108	GSE5616: AtGenExpress: Response to Phytophthora infestans	Scheel D et al. / Schildknecht B
	AT-00425	GSE20226: Arabidopsis thaliana/Phytophthora parasitica compatible interaction transcriptome	Attard A

Additional file 2. continued

insects	AT-00203	Silverleaf whitefly 2nd instar feeding on 7-week old Arabidopsis thaliana rosette leaves	Kempema LA, Cui X, Holzer FM, Walling LL
	AT-00250	GSE16497: Arabidopsis thaliana gene expression changes upon treatment with green peach aphid saliva	Jander G et al. / de Vos M
nemato-des	AT-00024	Plant gene expression associated with susceptibility to nematodes	Peter Edward Urwin
	AT-00121	Microarray analysis of Arabidopsis roots that have been infested with the root knot nematode Meloidogyne incognita	CG Taylor, E Nielsen, DP Schachtman, U. Hammes
Flg22	AT-00107 * (Figure 4F)	GSE5615: AtGenExpress: Response to bacterial-(LPS, HrpZ, Flg22) and oomycete-(NPP1) derived elicitors	Brunner F et al. / Schildknecht B
	AT-00253 *	E-NASC-76: Expression profiling of seedlings treated with oligogalacturonides (OGs) and Flg22	Dewdney J / Schildknecht B
	AT-00391 *	GSE17464: Arabidopsis mutants treated with flg22, Alternaria brassicicola, Methyl jasmonate and Pst DC3000 bacteria	Bari R et al. / Jones J
	AT-00392	GSE17479: Effect of auxin signaling on flg22 response	Robert-Seilaniantz A et al. / Jones JD
syringolin A	AT-00325 * (Figure 4G)	E-MEXP-739: Transcriptional changes in powdery mildew infected Arabidopsis leaves undergoing syringolin-triggered hypersensitive cell death at infection sites	Michel K
	AT-00258 *	Transcriptome changes in Arabidopsis mutant syl_404_bc2 after syringolin application	Michel K / Dudler R
	AT-00325 *	E-MEXP-739: Transcriptional changes in powdery mildew infected Arabidopsis leaves undergoing syringolin-triggered hypersensitive cell death at infection sites	Michel K
