# Supplementary material for: Plant plasma membrane-bound staphylococcal-like DNases as a novel class of eukaryotic nucleases
Source: BMC Plant Biol. 2012 Oct 26;12:195. doi: 10.1186/1471-2229-12-195 (PMC3505149; doi:10.1186/1471-2229-12-195)
Supplement: Additional file 3 — Plasma membrane localization of CAN1 and CAN2 nucleases in leaf protoplasts. [file 1471-2229-12-195-S3.pdf]

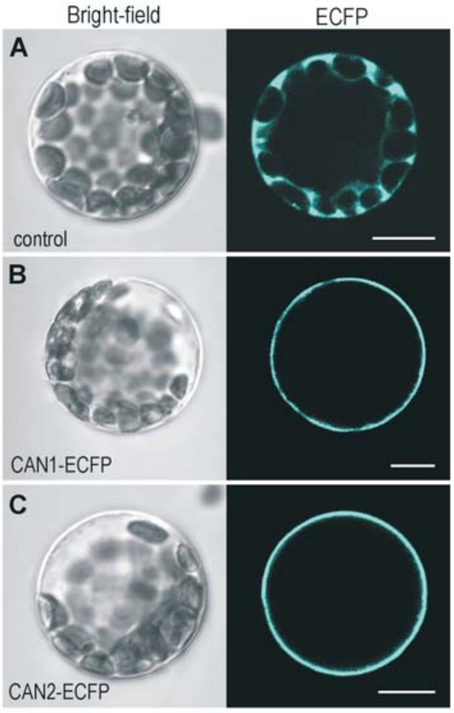

Additional file 3. Plasma membrane localization of CAN1 and CAN2 nucleases in leaf protoplasts. ECFP alone and ECFP-fusion proteins were transiently expressed in protoplasts from isolated leaf mesophyll cells. The bright-field images are shown in the left part of each panel and corresponding confocal laser scanning images are on the right. (**A**) Protoplast transformed with an empty vector (pSAT6A-ECFP) as a control. (**B**) Expression of the CAN1-ECFP fusion construct. (**C**) Expression of the CAN2-ECFP fusion construct. The scale bar indicates 10 μm.
