# Supplementary material for: Plant plasma membrane-bound staphylococcal-like DNases as a novel class of eukaryotic nucleases
Source: BMC Plant Biol. 2012 Oct 26;12:195. doi: 10.1186/1471-2229-12-195 (PMC3505149; doi:10.1186/1471-2229-12-195)
Supplement: Additional file 4 — The plasma membrane localization of CAN nucleases by fluorescence microscopy. [file 1471-2229-12-195-S4.pdf]

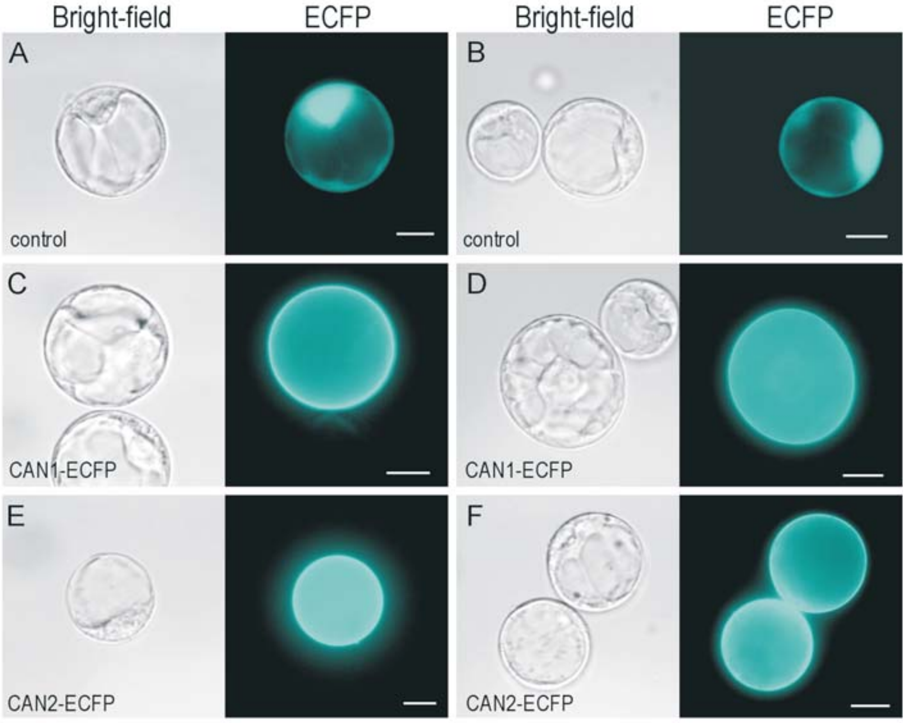

Additional file 4. The plasma membrane localization of CAN nucleases by fluorescence microscopy. ECFP alone and ECFP-fusion proteins were transiently expressed in *Arabidopsis* root cell protoplasts. The bright-field images are shown in the left part of each panel and corresponding ECFP fluorescence images are shown on the right. Two examples from each experiment are shown. (**A-B**) Protoplasts transformed with an empty vector (pSAT6A-ECFP) as a control. (**C-D**) Expression of the CAN1-ECFP fusion constructs. (**E-F**) Expression of the CAN2-ECFP fusion constructs. The scale bar indicates 10 μm.
